# Activating Mutation in the Receptor Tyrosine Kinase FLT3 with Clinicopathological Relevance in Canine Mast Cell Tumors

**DOI:** 10.1155/2022/9509900

**Published:** 2022-08-28

**Authors:** Nawin Manachai, Kasem Rattanapinyopituk, Piyanoot Fonghem, Panrawee Phoomvuthisarn, Shingo Nakahata, Kazuhiro Morishita, Anudep Rungsipipat

**Affiliations:** ^1^Center of Excellence for Companion Animal Cancer, Department of Veterinary Pathology, Faculty of Veterinary Science, Chulalongkorn University, Bangkok 10330, Thailand; ^2^Department of Companion Animals, Faculty of Veterinary Medicine, Chiang Mai University, Mae Hia, Muang, Chiang Mai 50100, Thailand; ^3^Department of Veterinary Surgery, Faculty of Veterinary Science, Chulalongkorn University, Bangkok 10330, Thailand; ^4^Division of Tumor and Cellular Biochemistry, Department of Medical Science, Faculty of Medicine, University of Miyazaki, Miyazaki 889-1692, Japan

## Abstract

Recent research has focused on the receptor tyrosine kinase (RTK) KIT which is involved in the pathogenesis of canine mast cell tumors (MCT). However, the role of other RTKs in this neoplasm remains unclear. The present study aimed to determine the frequency of FLT3 mutations and to evaluate the mutational status and clinicopathological relevance of canine MCT patients. There were a total of 20 cases that were cytologically and histopathological diagnosed as canine MCTs; genomic polymerase chain reaction (PCR) and Sanger sequencing were used to identify mutations. For the juxtamembrane (JM) domain, the FLT3 14/15 primer pair was used to investigate exon 14/15 loci. Based on genomic PCR amplification of exon 14/15 and 20 of the FLT3 gene and Sanger sequencing of 20 cases of canine MCTs, the overall frequency of FLT3 mutation in canine MCTs was 75%. The majority of FLT3 mutations (70%) were internal tandem duplications (ITD) of the JM domain, while one case arose from deletion mutations of the tyrosine kinase domain (TKD). However, double mutations were not observed in this study. Furthermore, there is also clinicopathological relevance to MCT dogs carrying FLT3-ITD mutations, showing a tendency toward leukocytosis due to neutrophilia, and resembling human acute myeloid leukemia (AML) with FLT3-ITD mutations. A subset of MCTs with FLT3-ITD mutations, showing an enhanced signal of phosphorylated ERK1/2 identified by immunoblotting, suggests that an activating mutation may be driven by a distinct signal of the ERK pathway. Our results indicate that FLT3-ITD mutation is an oncogenic driver of canine MCTs, and that it shares some clinicopathologic features with human AML. These findings may offer new opportunities for further studies on canine mast cell tumorigenesis and a novel therapeutic target for canine MCT cases harboring FLT3-ITD mutations.

## 1. Introduction

Aberrant activation of receptor tyrosine kinases (RTKs)—a crucial cancer-associated signaling molecule—is contributed via four main mechanisms: gain-of-function mutations, genomic amplification, chromosomal rearrangements, and/or autocrine activation. These mechanisms result in alteration of the kinase activity and allow malignant transformation [1, 2]. Activation of the mutation of the receptor tyrosine kinase (RTK) c-KIT and related signaling partners has been described as an oncogenic driver in the molecular pathogenesis of various types of human malignancies, including mast cell activation disorders, gastrointestinal stromal tumors, melanomas, and acute myeloid leukemia (AML) [3–5]. Accordingly, canine mast cell tumors (MCTs) are one of the most common skin tumors in dogs, representing 16–21% of all canine cutaneous neoplasms [6], characterized by abnormal expansion and accumulation of neoplastic mast cells in different tissues [7]. In addition, canine MCTs show extremely variable biologic behavior, from indolent to highly aggressive [8]. Accumulating evidence suggests that oncogenic activation by a gain-of-function mutation in KIT, accompanying both canine cutaneous MCTs and human systemic mastocytosis, includes internal tandem duplications (ITD) of exon 11 and the insertion or deletion of exons 8, 9, 11 or 17 [9–12], which then suggests KIT as an attractive therapeutic target for mast cell neoplasms. To date, tyrosine kinase inhibitors targeting KIT, such as masitinib and toceranib, have led to dramatic clinical responses in a subset of canine MCT patients, inhibiting the proliferation of neoplastic mast cells. However, the efficacy of KIT inhibitors in dogs with MCTs has been limited by several factors resulting in therapeutic resistance or relapse [13–15], which MCTs might be driven by additional somatic mutations.

It is well known that mast cells derived from multipotent hematopoietic progenitor cells of the bone marrow are similar to other cells in the myeloid lineage [16, 17]. Thus, mast cell malignancies are presumed to arise from clonal disorders of hematopoietic cells, resembling human myeloid neoplasm and/or myeloproliferative disorders [7, 18]. This indicates that malignant transformations by somatic mutations occurring in genes frequently mutated in myeloid malignancies should therefore be regarded as additional molecular features that may contribute to the pathogenesis of mast cell neoplasm in dogs. In particular, the FMS-like tyrosine kinase 3 (FLT3) is a transmembrane with a ligand-activated receptor tyrosine kinase along with the class III family of RTKs, as well as a c-KIT receptor that plays an essential role in normal and malignant hematopoiesis [19, 20]. Mutations of FLT3 are the most frequent genetic lesions, found in approximately 20 to 30% of newly diagnosed AML cases consisting of ITD mutations within the juxtamembrane (JM) domain and with a minor subset consisting of point mutations in the tyrosine kinase domain (TKD) [19, 21, 22]. A constitutively active TKR is displayed by both mutations in a ligand-independent manner, leading to alterations in normal hematopoiesis [19, 23, 24]. The ITD of the JM domain of FLT3 (FLT3-ITD) is the most frequent and significant driver of mutation for human AML patients; it demonstrates a high tumor burden and confers an unfavorable prognosis [23, 25, 26]. However, the frequency of these mutations and the clinical relevance of canine MCTs have not yet been characterized.

In the present study, we aimed to identify the FLT3 mutation of canine MCTs. Furthermore, we were able to evaluate the clinicopathological characteristics of various parameters of canine MCTs with respect to FLT3 mutation status.

## 2. Materials and Methods

A total of 20 cases, cytologically diagnosed as canine MCT were prospectively enrolled in the present study. After appropriate surgical excision as part of routine therapeutic procedures, tissue samples from all MCT-bearing dogs were submitted to the Department of Pathology, Faculty of Veterinary Science, Chulalongkorn University. All patients were pathologically confirmed, based on the two-tier histologic grading system for canine cutaneous MCTs [27]. All methods were carried out in accordance with relevant guidelines and regulations, and the sampling procedure was approved by the Chulalongkorn University Animal Care and Use Committee.

### 2.1. Clinicopathological Features

Clinical information for each case included the age, sex, and histologic grading of canine MCTs. The diagnostic workup at the initial diagnosis (including a complete blood count (CBC), mastocytemia, and serum biochemical profile) was evaluated in all MCT-bearing dogs.

### 2.2. Genomic DNA Extraction

Genomic DNA was extracted from 20 canine MCT tissue samples using the QIAGEN kit. According to the manufacturer's protocol (QIAamp DNA mini kit, QIAGEN, Germany), the quantity of extracted DNA was determined using a Nanodrop Lite Spectrophotometer (Thermo Scientific, MA, USA).

### 2.3. Mutation Analysis of *FLT3*

Genomic polymerase chain reaction (PCR) and Sanger sequencing were used to identify the mutations. For the JM domain, the FLT3 14/15 primer pair was used to investigate exon 14 through 15 loci. Moreover, the FLT3 20 primer pair was used for amplification of exon 20 corresponding to the TKD as previously described [28]. The primer sequences are listed in Table 1. PCR reactions were performed in 20 *µ*l reaction, containing 2 *µ*l buffer (10X), 1.6 *µ*l deoxyribonucleic phosphate (25 mM), 0.5 *µ*l of primer (20 ng/*µ*l) and 0.1 *µ*l (5 U/*µ*l) *Taq* DNA polymerase (Takara, Japan), and diluted to volume with MilliQ water. Thermal cycling programs were as follows: initial denaturation at 95 C for 5 minutes, 35 cycles of denaturation at 95 C for 30 seconds, annealing at 55 C for 30 seconds, followed by final extension at 72 C for 5 minutes. The amplified PCR products were visualized on 1.5% agarose gel electrophoresis to confirm their quality and amplicon size, and were further purified using a Qiaex II gel purification kit (Qiagen, Valencia, CA), following the manufacturer's instructions. The purified PCR samples were submitted for Sanger sequencing using the same primers used during amplification. Sequencing results were aligned to NCBI reference sequences based on the *Canis lupus familiaris* FMS-related receptor tyrosine kinase 3 (FLT3) gene.

### 2.4. Cloning and DNA Sequencing

To verify ITD mutation, FLT3 DNA fragments were excised for DNA purification. The DNA was purified by using a Qiaex II gel purification kit (Qiagen, Valencia, CA) according to the manufacturer's protocol. The purified DNA fragments were subcloned into pTA2 vectors using a TA cloning kit (Target Clone 0811, Toyobo, Japan) and were subsequently transformed into competent *Escherichia coli* cells according to the manufacturer's instructions. White colonies were isolated and cultured in lysogeny broth. Plasmids were puriﬁed using a QIAprep Spin Mini Kit (QIAgen, Crawley, West Sussex, UK) and sequenced in both directions using T7 and M13 primers.

### 2.5. Immunoblotting Analysis

Total protein lysate and western blot were performed as previously described [29]. Sodium dodecyl polyacrylamide gel electrophoresis and electroblotting were performed using primary antibodies, including anti-total ERK1/2 and phospho-ERK1/2 antibodies purchased from Cell Signaling Technologies (Beverly, MA). Secondary antibodies were horseradish peroxidase-conjugated anti-rabbit IgG (P0339; Dako, Denmark). Immunoreactive bands were developed using enhanced chemiluminescent reagents (Roche, Mannheim, Germany). Beta-actin housekeeping gene served as an internal control.

### 2.6. Statistical Analysis

Univariable analysis was performed by chi-square test to examine the difference between MCT-bearing dogs with FLT3-ITD mutations and MCT-bearing dogs with wild-type FLT3 (FLT3-WT) stratified by sex, age, and histological grading. Additionally, the Mann-Whitney *U* test was used to compare hematological parameters. All tests were two-tailed; *P* values < 0.05 were considered statistically significant. Tests were carried out with GraphPad Prism 6.0 (GraphPad Software, San Diego, CA, USA).

## 3. Results

Case characteristics were obtained from 20 dogs diagnosed with MCTs. The age, sex, and histologic grading for each case are shown in Table 2. The median age at the time of diagnosis for all MCT cases was 10.5 years (range 4–17). Of the 20 MCT-bearing dogs, 12 were intact males, 2 were castrated males, 4 were intact females, and 2 were spayed females. Moreover, following the two-tier histologic grading system for canine cutaneous MCTs [27], 9 dogs (45%) were classified as having low-grade MCTs and 11 dogs (55%) were classified as having high-grade MCTs.

### 3.1. Detection of FLT3 Mutations in Canine Mast Cell Tumor DNA Samples

To determine the frequency of FLT3 mutations, we examined DNA isolated from 20 surgically resected canine MCTs and used end-point PCR and Sanger sequencing to evaluate the mutational status of exons 14-15, the ITDs of the JM domain, and 20 TKD mutations.

**Figure 1:**
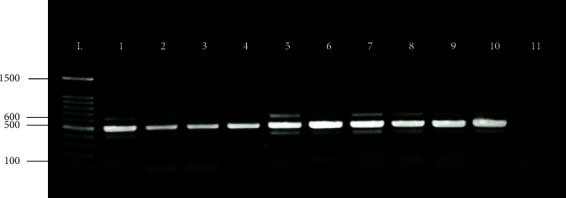
Detection of FLT3 ITDs in MCT-bearing dogs. FLT3 PCR products, obtained using the primers spanning exons 14 and 15, were analysed by agarose gel electrophoresis. A single band of 500 bp was obtained in FLT3-wild type MCT cases (lanes 3, 4, and 6), while an extra-band above 500 bp was shown in mutated samples (lanes 1, 2, 5, 7, 8, 9, and 10). Lane 1 - DNA ladder (100 bp); Lane 11 - negative PCR control (water).

For exon 14/15 of the JM domain, a single band at the 500 bp amplicon was detected while examining DNA isolated from FLT3-WT MCT-bearing dogs (Table 2, Cases 3, 4, 6, 16, 17, and 19). Representative examples are shown in Figure 1 (lanes 3, 4, and 6). In the case of the FLT3-ITD mutations, there was an additional band, that was higher than 500 bp and was identified at high frequency in 14 of 20 (70%) MCT-bearing dogs (Figure 1, lanes 1, 2, 5, 7, 8, 9 and 10). The FLT3-ITD mutation is an in-frame 100–120 bp duplication. The direct sequencing of purified clones (exons 14-15) showed the absence of point mutations and the presence of in-frame duplications of 100–120 bp. Following this, direct sequencing of FLT3 exon 20 was performed. The results of the FLT3 mutation analysis for each patient are shown in Table 2. Only one case was identical to MCT with the KIT mutation (Case 2) and there were only two cases of KIT mutation from all MCT-bearing dogs in this study.

### 3.2. Relationship between FLT3 Mutation Status and Clinicopathological Characteristics

**Figure 2:**
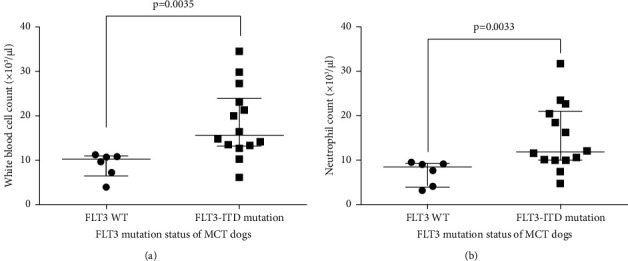
Difference in white blood cell (WBC) (a) and neutrophil (b) counts between. FLT3- WT and ITD mutation groups of MCT dogs. Data are presented as dot plots analysis of total WBC counts (a) and neutrophil counts (b) of canine MCT patients between FLT3-WT (*n* = 4) and FLT3-ITD mutation of JM domain (*n* = 16).

**Figure 3:**
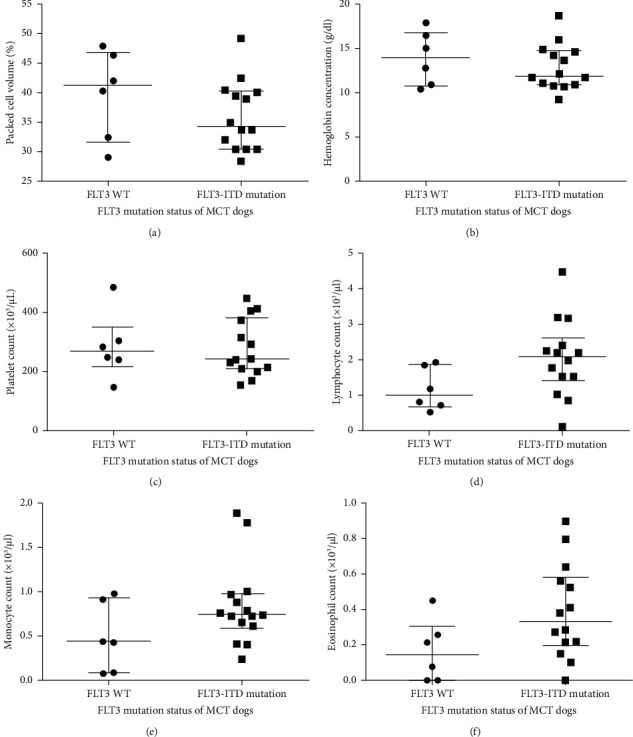
Hematological parameters in FLT3-WT and FLT3-ITD mutated dogs. Dot plots showing packed cell volume (a), haemoglobin concentration (b), platelet count (c), lymphocyte count (d), monocyte count (e), and eosinophil count (f) data of FLT3-WT (*n* = 6) and FLT3-ITD mutated (*n* = 14) dogs. No significant differences were observed between the two groups considering all haematological parameters.

Among the 20 dogs with cutaneous MCTs, 15 (75%) had mutations, 14 (70%) of which were ITD mutations, and 1 (9%) of which were deletion mutation of TKD. However, double mutations were not identified in this study. Regarding the high prevalence of MCT-bearing dogs showing FLT3-ITD mutation, these patients were divided into two groups: an FLT3-WT group and an FLT-ITD mutation of the JM domain group. Six (30%) patients were assigned to the FLT3-WT group and 14 (70%) were assigned to the FLT3-ITD mutation group. According to the univariable analysis, there was no association between sex, age, or histological grading and FLT3-ITD mutation status, as shown in Table 3. As part of the routine diagnostic workup, the complete blood count of all animals was assessed. Interestingly, canine MCT cases harboring FLT3-ITD mutations demonstrated significantly higher white blood cells (WBC) and absolute neutrophil counts in comparison to the FLT3- WT group at the time of presentation, as displayed in Figure 2(a) and 2(b). However, there was no significant difference in other hematological parameters including packed cell volume, hemoglobin levels, platelet, lymphocyte, monocyte, and eosinophil counts (Figure 3). Only 2 (10%) MCT-bearing dogs had circulating mast cells, which were identified using buffy-coat smears stained with toluidine blue. The clinico-hematologic characteristics of these patients are summarized in Table 3. The issue of follow-up data was not available due to the limitations of the study.

### 3.3. Activation of Downstream Signaling Molecules Detected by Immunoblotting

**Figure 4:**
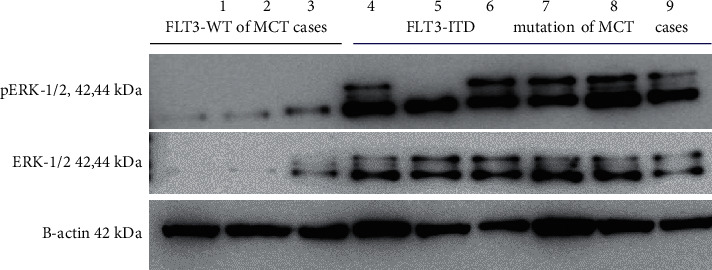
Canine MCT FLT3-ITD mutation activates ERK signaling. Total ERK1/2 and phosphorylated ERK1/2 expression in MCT was assessed by immunoblot analysis. Three cases of MCT-bearing dogs with FLT3-WT and six cases of MCT-bearing dogs with FLT3-ITD mutation was represented as indicated in the figure.

The activation of potential downstream mediators of FLT3 tyrosine kinase receptors was examined in order to explore FLT3 mutant signaling in nine cases of canine MCT. As reported in human FLT3-ITD AML, the activating mutation of FLT3 receptors enhances leukemic cell proliferation and resistance to apoptosis in a ligand-independent manner, achieved by constitutive activation of the RAS-ERK signaling pathway [30, 31]. We analyzed ERK protein expression using immunoblotting. Interestingly, ERK was mostly activated in the FLT3-ITD MCT group, which was detected by phosphorylation of ERK1/2 (pERK1/2) (Figure 4). This finding suggests that FLT3-ITD mutations may play an important role in activating tyrosine kinase-driven mast cell tumorigenesis in dogs via the RAS-ERK signaling pathway, in correspondence with human AML with FLT3-ITD mutations.

## 4. Discussion

The identification of additional somatic mutations is a molecular aberration that may not only provide information on the biological behaviors and prognosis of canine MCTs, but could also have an impact on therapeutic decisions. The phenotype and clinical variants of mast cell neoplasm are likely to be the result of a distinct mutational status in various malignant subclones of the myeloid lineage [18, 32]. Additionally, in human myeloid malignancies, including AML, chronic myeloid leukemia, and particularly mast cell leukemia, the leukemia-initiating cells are considered to occupy the CD34-positive subpopulation of the malignant clone [33–35]. Taken together, we propose that canine MCTs are clonal of myeloid-derived disorder and additional somatic mutations may occur. Further, the identification of these mutations may initiate new insight into the molecular characteristics and clinical importance of this neoplasm. This study is the first to report that FLT3 mutations occur in canine mast cell tumors using PCR analysis of genomic DNA and a hot-spot sequencing approach on a cohort of 20 MCT cases. Interestingly, a high frequency of in-frame mutations in FLT3-ITD of exon14 was identified, representing 70% (14/20) of the MCT-bearing dogs examined. This is also the most frequent type of somatic mutation in human AML [19, 21]. Although the prevalence of FLT3 mutations is high in human AML, it is not presented in systemic mast cell disease [19].

FMS-like FLT3 is a member of class III RTKs that plays a key role in normal hematopoiesis, which is tightly expressed by the CD34-positive fraction of hematopoietic progenitor cells [19, 36], which then means the alteration of the FLT3 receptor plays an important role in leukemogenesis [19, 37]. Activation of FLT3 by somatic mutation is the most frequently identified type of genomic lesion in human AML. One-third of AML patients have FLT3 mutations, and this mutation is associated with internal tandem duplication (ITD) in the JM domain of FLT3, promoting hyperactivation of tyrosine kinase signaling pathways coexisting with the expansion of the leukemia cell population [24, 25]. Currently, the high prevalence of FLT3-mutated genes in canine MCT patients is the effect of ITDs with sizes ranging from 15 bp to more than 400 bp, localized in the JM domain of the FMS-related RTK [38]. Additionally, in all ITD mutations of the FLT3 gene, agarose gel electrophoresis analysis demonstrated the presence of both mutated and wild-type alleles, indicating mutation heterozygosity in correspondence with previous reports of canine leukemia patients and cell lines [28, 39].

In humans, AML patients with FLT3-ITD mutations are significantly older than those without FLT3 mutations [40]. This suggests an association between FLT3 mutation status and increased frequency of mutation due to acquired somatic mutations in this gene. However, in the present study of canine MCT cases, there was no statistically signiﬁcant relationship between the presence of the mutation and the age of MCT-bearing dogs (*p*=0.78). The relationship between clinical relevance and the FLT3 mutation status of human AML has been discussed in several studies [19, 23, 39]. From a clinical perspective, AML patients with FLT3-ITD mutations have been associated with poor prognoses, increased white blood cell count at diagnosis, and an increased risk of relapse [25, 41]. Moreover, WBC count is also the most important prognostic factor in acute promyelocytic leukemia (APL), and there are poorer outcomes for patients presenting with high WBC counts [42, 43]. Here we describe for the ﬁrst time a univariate analysis demonstrating that WBC count is a significant clinicopathological factor in canine MCT patients, resembling human AML and APL with FLT3-ITD mutations. However, the basis for the elevated WBC counts in canine MCT patients with ITD mutations is not fully understood. This phenotype can be explained by one of two hypotheses. First, considering the two-hit model of leukemogenesis, FLT3-mutated genes are described by class I mutations, which confer a growth advantage along with protection from apoptosis [44]. Similar evidence has been reported in an *in vivo* study. A knock-in mouse model by inserting an internal tandem duplication mutation into the JM domain of murine FLT3 developed the myeloproliferative disease (characterized by splenomegaly and leukocytosis with myeloid lineage cell expansion) by inserting an ITD mutation into the JM domain of murine FLT3 [45]. Second, another possibility is that neoplastic mast cells can induce both local and systemic inﬂammatory responses [46, 47]. A recent study demonstrated that high-grade canine MCTs had higher ratios of leukocyte subsets in peripheral blood [47]. Altogether, our findings suggest the idea that inflammation may play a role in the pathogenesis of canine MCTs and drive the emergence of mutant clones of neoplastic mast cells to promote tumor progression.

According to guidelines for AML classification established by World Health Organization (WHO), genetic mutation of FLT3 with ITD as a molecular marker reveals an unfavorable prognosis, with an increased risk of relapse and shorter overall survival compared with AML patients without the mutation [25, 48]. However, the prognostic impact of FLT3 mutations in MCT canine patients was not presented in this study. These data are important and could possibly lead to predictions of clinical outcomes, including disease progression, overall survival, and response to various treatments, which would then require further investigation with a larger cohort. The mechanism of activation of mutations of FLT3 by the ITD has been well elucidated by *in vitro* and *in vivo* studies [19, 22, 49]. Loss of the autoinhibitory function of the activation loop, with subsequent constitutive activation of tyrosine kinase and its downstream oncogenic signaling pathways, including JAK-STAT, RAS-ERK, and PI3K-AKT pathways have all been shown to be involved [19, 20]. Similarly, hyperactivation of the RAS-ERK pathway as assessed by anti-phospho-ERK1/2 immunoblotting was shown in canine leukemia with the presence of FLT3-ITD mutations [39]. In this study, immunoblot assay revealed a subset of canine MCTs harboring ITD with increased phosphorylation of ERK1/2 levels compared with canine MCTs with FLT3-WT. These ﬁndings support the idea that activation of mutations of FLT3 may play an important role in mast cell tumorigenesis via RAS-ERK mutant signaling pathway activation. The current understanding of their molecular events offers new insight into their role in the pathogenesis of canine MCTs and will aid in the management of MCT carrying FLT3-ITD patients.

Until now, there have been only two tyrosine kinase inhibitors against KIT, which are masitinib and toceranib. They have been approved to treat unresectable or disseminated MCT in dogs. However, in some patients with aggressive MCTs, the therapeutic efficacy of these inhibitors is partial, and relapses are frequently demonstrated [13–15]. Given the gain-of-function ITD mutations of FLT3 in the majority of canine MCTs in this study, it seems promising to use FLT3 as a molecular target for the development of small molecular inhibitors. Several FLT3 inhibitors, for example, lestaurtinib and sorafenib, have been developed and investigated in human myeloid neoplasm patients with FLT3-ITD mutations [21, 23, 25]. Recently, midostaurin, a potent FLT3 inhibitor, has been FDA-approved [50]. In a recent veterinary study, midostaurin was tested for the first time in combination with ibrutinib and midostaurin, and together they produced synergistic growth-inhibitory effects against canine mastocytoma cell lines, *in vitro* [51]. However, the mutational status of tyrosine kinase-associated genes was not established, which would have indicated the possibility of FLT3 inhibitors in providing a new target therapy for MCT patients with this mutation.

## 5. Conclusion

We first identified FLT3 gene mutations in canine MCTs. A high frequency of FLT3-ITD somatic mutations in dogs with neoplastic mast cells elevated the WBC count, along with an increase in neutrophil subpopulations, corresponding to human AML patients harboring FLT3-ITD mutations with high tumor burden and poor clinical outcomes. Furthermore, analysis of a subset of canine MCTs carrying these mutations reveals RAS-ERK mutant signaling pathway activation. These findings support the idea that mutation activation driven by activation of the downstream ERK signaling pathway can provide new insight into its role in the molecular pathogenesis of MCTs, and may offer novel opportunities for the use of rational targeted therapies for MCT dogs harboring FLT3 mutations.

## Figures and Tables

**Table 1:** Forward (F) and reverse (R) primer sequences for polymerase chain reaction with corresponding annealing temperature and their amplification products used to investigate mutations.

Genes symbol and primer sequence (5′⟶3′)	Exon	Annealing temp.	Amplification product (bp)
*FLT3*			
F: CCATTTCTGAGGGACTGC	14-15	55	500
R: GCCTTGAAACATGGCAAGC			
F: TCACCTGGAATTCCTACTGAAC	20	55	308
R: TGTACTACAGCGGTTGTGGAC			

**Table 2:** Clinical information and FLT3 mutation status of 20 dogs with mast cell tumour.

Case no.	Age	Sex	Histopathological grading (kiupel system)	FLT3 Exon 14/15 mutation	FLT3 Exon 20 mutation	WBC count (×10^3^/*µ*l)
1	12	M	Low	ITD	No	27.28
2	10	M	Low	ITD	No	29.80
3	10	F	Low	WT	No	11.25
4	8	M	High	WT	No	10..89
5	13	M	Low	ITD	No	12.76
6	8	M	High	WT	No	7.39
7	12	M	High	ITD	No	14.21
8	10	Fs	High	ITD	No	13.34
9	8	Fs	High	ITD	No	6.22
10	3	M	High	ITD	No	21.39
11	7	Mc	High	ITD	No	16.48
12	10	F	High	ITD	No	19.95
13	17	M	High	ITD	No	14.76
14	12	M	Low	ITD	No	13.62
15	13	M	Low	ITD	No	10.25
16	11	F	Low	WT	No	4.04
17	13	M	High	WT	No	9.70
18	12	M	Low	ITD	No	34.54
19	11	F	Low	WT	*M*	10.72
20	4	Mc	High	ITD	No	22.82

M = male, Mc = castrated male, F = female, Fs = spayed female, ITD = internal tandem duplication mutation, WT = wild-type, No = No mutation, *M* = mutation.

**Table 3:** Comparison of clinicopathological characteristics between FLT3-ITD mutation and FLT3-WT of JM domain from 20 MCT dogs at the time of diagnosis.

	Total	FLT3-WT	FLT3-ITD	*P*
No. of MCT dogs (%)	20	6 (30)	14 (70)	
Median age (years)	10.5	10.5	11	0.7897
Sex				0.1731
M	12	4	8	
Mc	2	0	2	
F	4	3	1	
Fs	2	0	2	
Histologic grading				0.7686
Low	9	3	6	
High	11	3	8	
Laboratory findings				
Median WBC count (×10^3^/*µ*l) (range)	13.19 (4.04–34.54)	10.21 (4.04–11.25)	15.62 (6.22–34.50)	^*∗*^0.0035
Median neutrophil count (×10^3^/*µ*l) (range)	10.05 (3.23–31.74)	8.41 (3.23–9.53)	11.80 (4.71–31.74)	^*∗*^0.0033
Median lymphocyte count (×10^3^/*µ*l) (range)	1.82 (1.5–4.47)	1.01 (0.55–1.94)	2.09 (0.15–4.47)	0.05
Median monocyte count (×10^3^/*µ*l) (range)	0.73 (0.08–1.88)	0.44 (0.08–0.99)	0.7 (0.24–0.188)	0.2269
Median eosinophil count (×10^3^/*µ*l) (range)	0.27 (0–0.9)	0.15 (0–0.45)	0.33 (0–0.9)	0.0752
Median PCV (%) (range)	36.9 (28.4–49.2)	41.15 (29–47.9)	34.3 (28.4–49.2)	0.3017
Median platelet count (×10^3^/*µ*l) (range)	249 (148–487)	270 (148–487)	244 (1560–4 36)	0.8374
Median hemoglobin (g/dl) (range)	12.45 (9.2–18.6)	13.95 (10.4–17.9)	11.9 (9.2–18.6)	0.5062
Mastocytemia (%)	2 (10)	1 (10)	1 (10)	

M = male, Mc = castrated male, F = female, Fs = spayed female, PCV = packed cell volume ^*∗*^Statistically significant.

## Data Availability

The *Canis lupus familiaris* reference gene was obtained from the National Center for Biotechnology Information Assembly database using the accession number NM_001020811.1. These data were derived from the following resources available in the public domain: National Center for Biotechnology Information and https://www.ncbi.nlm.nih.gov/nucleotide/NM_001020811.1?report=genbank&log$=nuclalign&blast_rank=8&RID=2AB9AY7D016.
